# Household-Level Coverage of Iron-Biofortified Beans in the Northern Province of Rwanda

**DOI:** 10.1016/j.cdnut.2023.100106

**Published:** 2023-05-27

**Authors:** Theogene Dusingizimana, Andrew Jones, Hilda Vasanthakaalam, Tomas Kjellqvist

**Affiliations:** 1 Department of Food Science and Technology, College of Agriculture, Animal Sciences and Veterinary Medicine (CAVM), University of Rwanda, Kigali, Rwanda; 2 Department of Nutritional Sciences, School of Public Health, University of Michigan, MI, United States; 3 School of Natural Sciences, Technology and Environmental Studies, Södertörn University, Huddinge, Sweden

**Keywords:** biofortification, iron, beans, coverage, Rwanda

## Abstract

**Background:**

Biofortification, the process of enhancing the micronutrient content of staple crops, is a nutrition-sensitive agricultural intervention with the potential to increase micronutrient intakes and improve health outcomes, especially among vulnerable populations. Although data are available on the number of farming households that grow biofortified crops, information on the coverage of biofortified foods in the general population is limited. Such information is critical to assess the performance of biofortification programs and guide decisions related to program implementation while ascertaining progress toward achieving expected impacts.

**Objective:**

This study aimed to assess the household coverage of iron-biofortified beans (IBBs) in rural areas of the Northern Province of Rwanda.

**Methods:**

We applied methods previously used to assess coverage in large-scale food fortification programs to develop coverage indicators for IBBs. These indicators were *1*) consumption of beans in any form; *2*) awareness of IBBs; *3*) availability of IBBs; *4*) consumption of IBBs (ever); and *5*) consumption of IBBs (current).

**Results:**

Of the 535 households surveyed, 98% consumed beans in any form and 79% were aware of IBBs. Among the 321 households that provided bean samples, only 40% of the samples were biofortified (as determined by a breeding specialist) and only 21% of respondents were able to correctly identify IBBs. Although 52% of households reported to be ever consuming biofortified beans, only 10% of households were currently consuming these beans.

**Conclusions:**

Despite relatively high awareness of IBBs among surveyed households, a few households currently consume IBBs, highlighting the need to explore strategies to promote consumption. More research is also required to investigate factors hindering the consumption of IBBs.

## Introduction

Micronutrient deficiencies remain a significant global public health challenge, affecting more than 2 billion people worldwide [1]. The most widespread micronutrient deficiencies globally are those of iron, zinc, selenium, vitamin A, and folate [2]. Iron deficiency is probably the most common micronutrient deficiency, and its most common consequence is iron deficiency anemia. In Rwanda, anemia affects 37% and 24% of children younger than 5 y and pregnant women, respectively [3]. The prevalence of anemia is particularly high among younger children, affecting 70% of children aged 6–8 mo and 64% of those aged 9–11 mo. Other micronutrient deficiencies are also prevalent. For example, Ritchie and Roser (2019) estimated that, in 2005, 35% of the Rwandan population had zinc intake below the physiological requirements [4]. Implications of these deficiencies on health and economic productivity are well documented. For example, iron deficiency anemia leads to cognitive impairment, increased risk of child and maternal morbidity and mortality, and low productivity at work [2]. Evidence suggests that these deficiencies can be partly attributed to inadequate intake of dietary micronutrients due to diets that are inadequate in terms of vitamin A, calcium, and iron [5,6].

To address micronutrient deficiencies, the Rwandan government adopted a multisectoral approach including nutrition-specific and nutrition-sensitive programs. Biofortification, a process for enhancing the micronutrient content of staple crops through plant breeding, is regarded as a promising, sustainable, and cost-effective food-based approach for delivering micronutrients to populations that may have limited access to diverse diets or commercially marketed fortified foods and supplements [7,8]. It is one of the nutrition-sensitive agricultural interventions implemented as part of the Rwandan government’s multisectoral strategy to address micronutrient deficiencies. Randomized controlled trials conducted in different countries have successfully demonstrated that consuming biofortified crops can significantly improve the micronutrient status of target populations. For example, studies conducted in Rwanda [9] and India [7] found that consuming iron-biofortified beans improved hemoglobin and total body iron stores.

The premise of biofortification is that if biofortified staple crops are widely grown and consumed by the target populations, their nutritional status will improve, which could lead to positive health and economic outcomes [10]. However, the effectiveness and impact of biofortification programs are not only determined by biological efficacy but also by high program coverage and effective implementation [11]. Bouis et al. [12] and Qaim et al. [10] argued that the success and potential impact of biofortification programs are largely determined by high coverage in terms of farmer adoption and coverage of biofortified crops. Unfortunately, reliable data and appropriate indicators on coverage of biofortification programs are often limited [13]. This represents an important area of investigation. The need to generate regular data on the coverage and utilization of nutrition programs, such as biofortification, as a means of tracking progress and enhancing evidence-informed decision making in program implementation has previously been highlighted [14,15].

Coverage, defined as the proportion of intended program beneficiaries that receive the program, is a useful indicator that can be used as a proxy for program performance and can serve to document the progress in the scale-up of interventions [16,17]. To date, 14 iron-biofortified bean varieties are grown in Rwanda. The first varieties of these beans were introduced to farmers between 2010 and 2012. Past studies on iron-biofortified beans (IBBs) in Rwanda have mainly focused on farmers’ adoption of IBBs [[18], [19], [20], [21]]. One of these studies [21] found that the adoption of one of the most widely disseminated IBB varieties can contribute to increased iron intake through increased consumption of iron-rich beans from own production among adopting households and that the adoption leads to an increased likelihood of selling IBBs, which benefits nonadopter households. Other studies have focused on iron bioavailability and retention [22], efficacy [9,23], as well as consumer acceptance and willingness to pay for IBBs [24]. Although studies on adoption provided important insights into the proportion of households that are growing biofortified beans, the studies do not provide enough information on other critical aspects such as awareness, consumption, and availability of biofortified crops. Such information is crucial because, although biofortified crops were introduced in Rwanda more than a decade ago, the crops are still relatively new to both farmers and consumers and, in many African countries, the crops have not been fully integrated into local food systems [25]. To our knowledge, only 1 study examined the coverage of biofortified crops in Rwanda. The study involved 242 households from one district (Musanze) in Rwanda and reported low consumption, i.e., 15% ever consumption and 10% current consumption, of IBBs [26]. The authors concluded that low coverage of biofortified crops was attributed to the bottlenecks in biofortification program implementation, more specifically low awareness and availability of biofortified foods [26])

The present study aimed to determine household-level coverage of IBBs for identifying areas of improvement to accelerate the progress and scaling-up of biofortification programs in Rwanda. The study also examined the extent to which IBBs are reaching the most nutritionally vulnerable households to explore potential inequalities in biofortification programs.

## Methods

### Survey design and participants

This study was a cross-sectional household-based survey. It was conducted as part of a larger interdisciplinary project implemented to improve women’s and children’s nutrition in the Northern Province of Rwanda. Administratively, Rwanda is divided into 4 provinces. Each province is divided into districts, which are in turn divided into sectors, cells, and villages – a village is the lowest administrative unit. For the current study, 3 (out of 5) districts of the province were selected based on similarities in agroecological characteristics including major crops grown and cropping system. Two districts were excluded because, one district, i.e., Musanze, is a largely an urban district, and another, i.e., Gakenke, was excluded because its agroecology replicates other rural districts in the province. To select participants, 2 sectors were first randomly selected from each of the 3 selected districts using a database from the main project. Next, 2 villages were randomly selected in each sector (12 villages in total). Then, 45 households were randomly selected in each village based on available resources. In total, 535 households were selected. The survey was conducted between August and September 2022. During this period, IBBs were expected to be available based on local planting and harvesting schedules in the districts.

### Data collection

Data were collected using a semi-structured questionnaire, administered to a female representative from the household aged 15–49 y. In the cases where there were more than 1 female aged 15–49 y in the household, the oldest was selected. The questionnaire was preprogrammed in both English and Kinyarwanda (local language) using Open Data Kit software to allow for data collection on mobile phones. Information on household adoption, awareness, purchase, and consumption of biofortified beans was collected, as well as data on household sociodemographic characteristics and crop and livestock production. A qualitative 24-h dietary recall instrument was also used to assess the dietary diversity of the index respondent. A team of 6 trained research assistants collected the data under the supervision of the first author who closely monitored the data collection and conducted daily checks of the completeness of the questionnaires to ensure the accuracy of the data. The questionnaire was pilot tested in another community with similar characteristics to the study population.

### Indicators of coverage of IBBs

We adapted previously developed indicators and followed the Tanahashi model [27] to assess coverage. We assessed coverage in terms of awareness, consumption, and availability of IBBs based on responses to relevant questions in the questionnaire. The first indicator, i.e., *consumption of beans (in any form)*, was based on the response to the question, “Do you consume beans in your household?” The second indicator, i.e., *awareness of the iron-biofortified beans*, was based on the response to the question, “Have you ever heard of iron-biofortified beans?” The third indicator, i.e., *the availability of iron-biofortified beans in the household*, was based on results from a sampling of household bean supplies. Briefly, during data collection respondents were asked if they had any beans in their households. A small amount of beans was collected from households that had them and agreed to part with them. If a household had more than 1 variety, respondents were asked to give a sample from the bean variety that they believed was iron-biofortified. In total, 321 bean samples (from 60% of the households) were collected and later visually analyzed by a bean breeding specialist to determine whether IBBs were available or not in the household. Thus, households that did not provide bean samples were excluded from the analyses for the “availability” indicator. The fourth indicator, i.e., *consumption of iron-biofortified beans (ever)*, was determined based on the response to the question, “Have you ever bought/received biofortified beans for eating?” The fifth indicator, i.e., *consumption of iron-biofortified beans (current)* was based on the response to the question, “Have you consumed biofortified beans in the last 7 days?”

### Identifying nutritional at-risk households

A household’s nutritional risk was determined based on 2 indicators, namely, the Multiple Poverty Index (MPI) [28] and the Minimum Dietary Diversity for Women (MDD-W) [29] indicators.

The MPI is a measure that takes a value between 0 and 1 and consists of a weighted sum of 3 dimensions, each contributing one-third of the sum: education, health, and living standards [28]. For the current study, the education dimension consisted of 2 indicators, namely, the education of the household head and school attendance of school-age children. Each indicator of the education dimension was worth one-sixth of the sum. The health dimension also consisted of 2 indicators, namely, the caregiver’s nutrition (proxied by the MDD-W indicator) and access to health services (proxied by household coverage of health insurance). Each indicator was worth one-sixth of the sum. The living standards dimension consisted of 6 indicators each worth one-eighteenth of the sum. These included household’s access to electricity/solar panels, type of flooring material, type of cooking fuel, the safety of drinking water, type of latrine, and ownership of key assets (including radio, television, personal computer or tablet, mobile phone, bike, motorbike, iron, and mattress). For all dimensions, each indicator was given a value of 0 if the response corresponded to the characteristics of a nonpoor household; otherwise, the value was one-sixth or one-eighteenth according to the indicator. A household was classified as being poor if the MPI score was ≥ 0.33, and it was classified as nonpoor if the MPI score was < 0.33 (on a scale of 0–1).

The MDD-W indicator was constructed following the MDD-W measuring guide [29]. It is an indicator of whether or not a woman has consumed at least 5 out of 10 defined food groups the previous day or night. The 10 food groups considered are *1*) starchy staple foods (grains, white roots and tubers, and plantains); *2*) pulses (beans, peas, and lentils); *3*) nuts and seeds; *4*) milk and dairy products; *5*) meat, fish, and poultry; *6*) eggs; *7*) dark green leafy vegetables; *8*) vitamin A-rich fruits and vegetables; *9*) other vegetables; and *10*) other fruits. The MDD-W indicator was developed and validated as a proxy for better micronutrient adequacy of a population diet [30]. A household was classified as being nutritionally at risk if the count of food groups consumed was <5, and it was classified as nutritionally not at risk if the count of food groups consumed was ≥5.

### Ethical approval

This study was conducted as part of a larger study that had received ethical clearance from the National Health Research Committee and the University of Rwanda’s College of Medicine and Health Sciences (reference numbers: NHRC 2020//PROT/047 and 295/CMHS IRB/2022, respectively). Before data collection, district offices, and community leaders where data were collected were informed to facilitate access to the community. Informed verbal consent was obtained from each respondent before data collection.

### Data analysis

Descriptive statistics, including means (ranges) for continuous data and percentages for categorical data, were used to describe the socioeconomic characteristics of participants. The different coverage indicators were expressed as the proportions (plus 95% CIs) of households that were aware of or consumed (ever of current) IBBs or provided bean samples that were judged iron biofortified. Chi-square tests were conducted to assess the significance of differences between nutritionally at-risk households and nutritionally not-at-risk households. Data analysis was conducted using SPSS version 25.0 (IBM Corp.).

## Results

The main characteristics of the surveyed households are shown in Table 1 [31]. On average, households had 4 members and most of the households had a male household head. Although most (74%) of the households had access to a safe drinking water source, only 39% of the households had access to adequate sanitation. Most households (77%) owned land for crop production. Beans and maize were crops grown by 82% and 50% of the households, respectively. Livestock ownership was also high, with 79% of households keeping at least 1 domestic animal. The proportion of households that kept cattle, sheep, and poultry were 58%, ∼22%, and ∼21%, respectively. Based on the 2 indicators of nutritional risks, 57% of the households were classified as multidimensionally deprived (MPI ≥ 0.33) and 51% did not meet the MDD-W indicator (i.e., diet diversity score < 5).

**TABLE 1:** Socioeconomic characteristics of respondents/households

Socioeconomic characteristics	*n* (%) or Mean [range]
Gender of household head (male)	470 (87.9)
Respondent age (y)	33 [15–49]
Household size	4 [2–12]
Household has access to safe drinking water source^1^	397 (74.2)
Household has access to adequate sanitation^2^	213 (39.8)
Household owns agricultural land	415 (77.6)
Crops grown by households^3^	
Beans (any)	441 (82.4)
Iron-biofortified beans (ever)^4^	157 (36.9)
Iron-biofortified beans (last season)^5^	57 (13.4)
Maize	267 (49.9)
Sweet potatoes	255 (47.7)
Irish potatoes	222 (41.5)
Green leafy vegetables	215 (40.2)
Sorghum	157 (29.3)
Cassava	81 (15.1)
Household owns any livestock^3^	421 (78.7)
Cattle	311 (58.1)
Goats	82 (15.3)
Sheep	115 (21.5)
Poultry	112 (20.9)
Respondent education (none or incomplete primary)	267 (49.9)
Household at risk of poverty^6^	306 (57.2)
Respondent with poor WDDS^7^	276 (51.6)

*N*=535; WDDS, Women Dietary Diversity Score.

^1, 2^ Defined according to the World Health Organization [31].

^4, 5^ Only participants who have heard of iron-biofortified beans were included in the analysis (*n* = 424).

3 Respondents provided multiple responses.

6 Defined as multidimensional poverty index ≥ 0.33.

7 Defined as a diet diversity score below 5.

**FIGURE 1:**
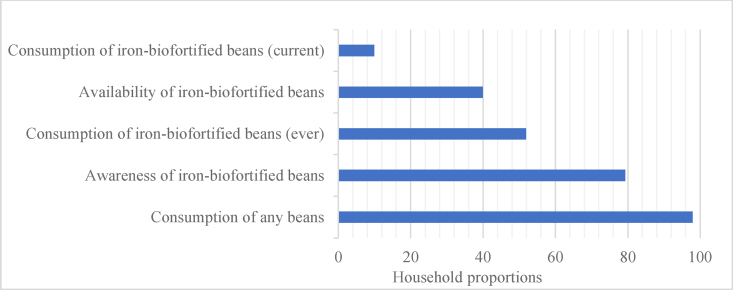
Indicators of coverage of iron-biofortified beans in Northern Province, Rwanda.

Figure 1 shows the 5 indicators of coverage of IBBs. Almost all surveyed households (98%) reported consuming beans (in any form), and 79% of the respondents were aware of IBBs. Respondents reported that they first had heard about IBBs most often from fellow farmers or farmer cooperatives (35.1%), radio or television (∼20%), or relatives/friends/neighbors (15.1%). Extension agents and agro-dealers were the least common source of information about IBBs. Of those that have ever heard of IBBs, 36% ever grew these beans, whereas only 13% had grown them in the previous cropping season. Despite high consumption of beans and awareness of IBBs, just over a half (51.9%) of the household respondents reported ever consuming IBBs in their households, and only approximately 10% of the households had consumed these beans in the past 7 d (see Supplementary Table 1 for the proportions and CIs). We also examined coverage between nutritionally at-risk and nutritionally not-at-risk households (Supplementary Table 2). Except for the awareness coverage indicator, all other indicators were not associated with nutritional vulnerability. The level of awareness was significantly higher among respondents with adequate MDD-W compared with respondents with inadequate MDD-W (83.4% vs. 75.4%, *P* = 0.027). Similarly, significantly more respondents from households that were multidimensionally nondeprived were aware of IBBs compared with respondents from multidimensionally deprived households (85.6% vs. 74.5%, *P* = 0.001). Household socioeconomic characteristics, including ownership of radio, mobile phone, bicycle, farmland, access to electricity/solar panel, and civil status were associated with awareness of IBBs. Consumption (current and ever) of IBBs was associated with civil status and radio ownership (Supplementary Table 3).

Results from the visual analysis of beans (Table 2) showed that approximately 40% of households had biofortified beans (as determined by bean a breeding specialist). Of these, 21% correctly reported that the type of beans they had were biofortified; 38% reported that the beans available in their households were nonbiofortified, whereas 41% did not know whether the kind of beans they had were biofortified or not.

**TABLE 2:** Biofortification status of collected bean samples

		Bean type as determined by a breeding specialist		
		Biofortified	Nonbiofortified	Total
Biofortification status as presumed by household respondents	Biofortified	27 (8.4%)	9 (2.8%)	36 (11.2%)
	Nonbiofortified	49 (15.3%)	104 (32.4%)	153 (47.7%)
	Do not know	53 (16.2%)	80 (24.9%)	132 (41.1%)
	Total	128 (39.9%)	193 (60.1%)	321 (100%)

## Discussion

The current study used previously developed theory-based indicators to assess the coverage of IBBs. These indicators may serve to better assess the performance of biofortification programs and to inform the scaling-up of these programs compared with other indicators that often focus on access to biofortified seeds/planting material coverage or on the number of households that grow biofortified crops [26,32,33]. Findings from the present study showed that beans featured prominently in the Rwandan diet and are an important crop grown by the surveyed households [34]. The results also confirm that beans have the potential to be used as a staple food vehicle for biofortification to address iron deficiency among children and women in Rwanda. Rwanda has one of the highest per capita consumption of beans in the world [35] and beans are often referred to as the meat of the poor because of their high protein content.

In the present study, high awareness coverage indicators among participants may reflect exposure to awareness creation activities to influence the behavior of households to grow and consume IBBs in Rwanda. Previous awareness campaigns to promote IBBs in Rwanda have used mass media and leveraged the power of local icons such as music stars to impart knowledge about the benefits of IBBs [36]. Awareness of new technology has been highlighted as one of the key prerequisite conditions for the adoption of agricultural technology [37,38] which, in the context of this study, can be leveraged to also enhance the utilization of a technology such as biofortification. Although further research is needed, our findings of a significantly higher awareness level among households with adequate minimum dietary diversity as compared with those with inadequate minimum dietary diversity, and among nondeprived households as compared with deprived households, tend to suggest that, in this study setting, awareness of IBBs may be influenced by household-level factors that also determine both dietary diversity and socioeconomic status. In our analysis, awareness of IBBs was found to be associated with many socioeconomic characteristics, including ownership of information-related assets (radio, mobile phone), access to electricity, land, and civil status.

Despite relatively high awareness of IBBs among the study participants, consumption coverage indicators were low, with the current consumption indicator being the lowest. These results suggest that many participants in our study were aware of the existence and, possibly, nutritional benefits of IBBs but, as discussed below, there may be other barriers to the consumption of these beans. Thus, strategies to strengthen efforts to increase awareness while bridging the gap between awareness and consumption are required. Our results on ever consumption coverage indicator are, however, higher than reported by Petry et al., in a study conducted in Musanze District, Rwanda, where only 15% of the households reported ever consuming IBBs. Both their and our findings were similar in terms of the current consumption coverage indicator [26]. The discrepancy between results on ever consumption reported in our study and those of Petry et al. may be due to differences in study settings and/or sample sizes. Our study included only rural farming households, whereas Petry et al.’s study focused on a more urbanized district and included both rural and urban participants. Both studies also differed in terms of sample sizes (242 households vs. 535 households). It had been projected that 15% of the population in Rwanda would be consuming biofortified crops in 2018 [32]. Unfortunately, the lack of research on household consumption of IBBs in Rwanda limits the comparability of data.

Previous studies in other countries have suggested that low consumption of biofortified crops and food products is linked to low awareness of the benefits of iron beans, particularly among rural consumers [39]. However, low consumption coverage indicators may be explained by other factors. For example, the value chains for most biofortified crops remain underdeveloped in many countries [25,39]. Some evidence from Rwanda also suggests that there is an unmet demand for biofortified bean seeds which partly results from weakness in the biofortified bean seed supply systems [34], and this may result in low availability, and hence consumption, of IBBs. In addition, low consumption may be due to household economic constraints. Available data indicate that, in Rwanda, the average retail price of IBBs is higher compared with local varieties [40]. At the time of data collection, the price of IBBs ranged between 1000 RWF and 1500 RWF per kilogram (i.e., 0.92 USD–1.38 USD) compared with 500 RWF–700 RWF (0.46 USD–0.92 USD) per kilogram for local bean varieties (data not shown). Research in low- and middle-income countries also shows that the high price of micronutrient-dense foods, including beans, often limits the demand and consumption of these foods [41].

The findings from this study also suggest that, although many efforts focus on increasing awareness of the nutritional benefits of IBBs, increased awareness alone may not be sufficient to incentivize households to substitute cheaper, nonbiofortified beans for IBBs. According to Qaim et al., even if the nutritional benefits of biofortified crops are appreciated by consumers, the willingness and ability to pay higher prices for biofortified foods may be limited, especially among the poor [10]. Thus, interventions to increase awareness and consumption of IBBs must be coupled with strategies to make the beans economically accessible to households, especially the poor who, when the price of nutritious foods increases, often prioritize less nutritious food commodities [41].

Although the availability and consumption coverage indicators were low, a large proportion of the study participants provided bean samples that the participants deemed were nonbiofortified or did not know the status but were judged iron biofortified. This is not surprising given that IBB varieties are not easily distinguishable from conventional varieties. IBBs are considered in the economics literature as *credence goods,* i.e., goods whose attributes may even ex-post be difficult to detect by consumers [42,43]. Thus, assuming that these participants donated samples from beans they were consuming at the time of the study, this would suggest that both the consumption and availability coverage indicators could have been higher than our current estimates. Therefore, caution in the interpretation of these results is required. Future field research to assess the coverage of IBBs should consider these findings.

By applying coverage indicators on consumption, awareness, and availability of IBBs, this study adds to the limited information required to inform the progress of biofortification program implementation. A limitation of the study is that it is based on data collected in one province, which limits the generalizability of the findings. In addition, only 45 households per village were selected due to limited resources. This could have led to high similarity among subjects and may have influenced our findings. Despite these limitations, the findings can still be applied broadly to studies that assess the coverage of biofortified crops in other settings where biofortified crops are being introduced.

The results of this study show that, despite relatively high awareness of IBBs among surveyed households, few households in the study area currently consume IBBs, highlighting the need to explore strategies to promote consumption. More in-depth research is also required to investigate factors hindering the consumption of IBBs.

## Author contributions

The authors’ responsibilities were as follows —TD, AJ, HV, and TK designed the research; TD conducted the research, performed statistical analysis, and wrote the first draft manuscript; and all authors contributed to the results interpretations, review and editing, and read and approved the final manuscript.

## Data availability

Data described in the manuscript and analytical code will be made available upon request pending application and approval by the corresponding author.

## Funding

This article is an output of the research program “Undernutrition – an interdisciplinary program focused on children and mothers in Rwanda,” a five-year collaboration between the University of Rwanda and five Swedish universities. We thank the Swedish International Development Agency (SIDA) for funding, the University of Rwanda for logistical support, and the University of Gothenburg, Lund University, Swedish University of Agricultural Sciences, Södertörn University, and Umeå University for their overall assistance and collaboration. The work also received additional funding through the Innovative Methods and Metrics for Agriculture and Nutrition Action (IMMANA) program, led by the London School of Hygiene and Tropical Medicine (LSHTM). IMMANA is cofunded with UK Aid from the UK government and by the Bill & Melinda Gates Foundation INV-002962/OPP1211308. The content, findings, conclusions, or recommendations expressed here are solely those of the authors.

## Author disclosures

The authors report no conflicts of interest.

## Declaration of interests

The authors declare the following financial interests/personal relationships which may be considered as potential competing interests: Theogene Dusingizimana reports financial support was provided by The Swedish International Development Agency (SIDA). Theogene Dusingizimana reports financial support was provided by Research Programme on Innovative Methods and Metrics for Agriculture and Nutrition Action (IMMANA), funded by the UK Aid from the UK government and by the Bill & Melinda Gates Foundation.

## References

[1] Muthayya S., Rah J.H., Sugimoto J.D., Roos F.F., Kraemer K., Black R.E. (2013). The global hidden hunger indices and maps: an advocacy tool for action. PLoS One.

[2] Bailey R.L., West K.P., Black R.E. (2015). The epidemiology of global micronutrient deficiencies. Ann. Nutr. Metab..

[3] National Institute of Statistics of Rwanda (NISR) (2020).

[4] Ritchie H., Roser M. (2019). https://ourworldindata.org/micronutrient-deficiency#anemia-in-pregnant-women.

[5] Umugwaneza M. (2017). https://repository.nwu.ac.za/bitstream/handle/10394/26417/%20Umugwaneza_M_2017.pdf.

[6] Grosshagauer S., Milani P., Kraemer K., Mukabutera A., Burkon A., Pignitter M. (2020). Inadequacy of nutrients and contaminants found in porridge-type complementary foods in Rwanda. Matern. Child Nutr..

[7] Bouis H.E., Saltzman A. (2017). Improving nutrition through biofortification: a review of evidence from HarvestPlus, 2003 through 2016. Glob. Food Sec..

[8] Meenakshi J.V., Johnson N.L., Manyong V.M., DeGroote H., Javelosa J., Yanggen D.R. (2010). How cost-effective is biofortification in combating micronutrient malnutrition?. An ex ante assessment, World Dev..

[9] Haas J.D., Luna S.V., Lung’aho M.G., Wenger M.J., Murray-Kolb L.E., Beebe S. (2016). Consuming iron biofortified beans increases iron status in Rwandan women after 128 days in a randomized controlled feeding trial. J. Nutr..

[10] Qaim M., Stein A.J., Meenakshi J.V. (2007). Economics of biofortification. Agric. Econ..

[11] Osendarp S.J.M., Martinez H., Garrett G.S., Neufeld L.M., De-Regil L.M., Vossenaar M. (2018). Large-scale food fortification and biofortification in low- and middle-income countries: a review of programs, trends, challenges, and evidence gaps. Food Nutr. Bull..

[12] Bouis H.E., Hotz C., McClafferty B., Meenakshi J.V., Pfeiffer W.H. (2011). Biofortification: a new tool to reduce micronutrient malnutrition. Food Nutr. Bull..

[13] Rodas-Moya S., Giudici F.M., Mudyahoto B., Birol E., Kodish S.R., Lachat C. (2022). Critical review of indicators, metrics, methods, and tools for monitoring and evaluation of biofortification programs at scale. Front. Nutr..

[14] (2015). The Global Panel on Agriculture and Food Systems for Nutrition, Biofortification: an agricultural investment for nutrition.

[15] Neufeld L.M., Baker S., Garrett G.S., Haddad L. (2017). Coverage and utilization in food fortification programs: critical and neglected areas of evaluation. J. Nutr..

[16] Boerma T., Requejo J., Victora C.G., Amouzou A., George A., Agyepong I. (2018). Countdown to 2030: tracking progress towards universal coverage for reproductive, maternal, newborn, and child health. Lancet.

[17] Health Information Systems Knowledge Hub (2012). Assessing health system performance using effective coverage – PubMed. Pac. Health Dialog..

[18] Nsengiyumva A., Mbabazi P., Kavoi M. (2017). Determinants of biofortified beans adoption in Nyagatare district. Eastern Province of Rwanda.

[19] Vaiknoras K., Larochelle C., Birol E., Asare-Marfo D., Herrington C. (2019). Promoting rapid and sustained adoption of biofortified crops: what we learned from iron-biofortified bean delivery approaches in Rwanda?. Food Policy.

[20] Larochelle C., Alwang J. (2022). Impacts of improved bean varieties adoption on dietary diversity and food security in Rwanda. Eur. J. Dev. Res..

[21] Vaiknoras K., Larochelle C. (2021). The impact of iron-biofortified bean adoption on bean productivity, consumption, purchases and sales. World Dev.

[22] Glahn R., Tako E., Hart J., Haas J., Lung’aho M., Beebe S. (2017). Iron bioavailability studies of the first generation of iron-biofortified beans released in Rwanda. Nutrients.

[23] Tako E., Reed S., Anandaraman A., Beebe S.E., Hart J.J., Glahn R.P. (2015). Studies of cream seeded carioca beans (*Phaseolus vulgaris L.*) from a Rwandan efficacy trial: in vitro and in vivo screening tools reflect human studies and predict beneficial results from iron biofortified beans. PLoS One.

[24] Oparinde A., Birol E., Murekezi A., Katsvairo L., Diressie M.T. (2015). Consumer acceptance of biofortified iron beans in rural Rwanda: experimental evidence. Washington, D.C. International Food Policy Research Institute (IFPRI). Report No. HarvestPlus Working Paper.

[25] Foley J.K., Michaux K.D., Mudyahoto B., Kyazike L., Cherian B., Kalejaiye O. (2021). Scaling up delivery of biofortified staple food crops globally: paths to nourishing millions. Food Nutr. Bull..

[26] Petry N., Wirth J.P., Friesen V.M., Rohner F., Nkundineza A., Chanzu E. (2020). Assessing the coverage of biofortified foods: development and testing of methods and indicators in Musanze, Rwanda. Curr. Dev. Nutr..

[27] Tanahashi T. (1978). Health service coverage and its evaluation. Bull. World Health Organ..

[28] Alkire S., Santos M.E. (2014). Measuring acute poverty in the developing world: robustness and scope of the multidimensional poverty index. World Dev.

[29] Martin-Prével Y., Allemand P., Wiesmann D., Arimond M., Ballard T., Deitchler M. (2015). https://www.fao.org/3/i4942e/i4942e.pdf.

[30] Martin-Prevel Y., Arimond M., Allemand P., Wiesmann D., Ballard T.J., Deitchle M. (2017). Development of a dichotomous indicator for population-level assessment of dietary diversity in women of reproductive age. Curr. Dev. Nutr..

[31] World Health Organization (2011). https://apps.who.int/iris/handle/10665/44584.

[32] Baral A. (2021). https://www.harvestplus.org/in-rwanda-sustained-success-for-iron-biofortified-beans/.

[33] Adekambi S.A., Okello J.J., Abidin P.E., Carey E. (2020). Effect of exposure to biofortified crops on smallholder farm household adoption decisions: the case of orange-fleshed sweetpotato in Ghana and Nigeria. Sci. Afr..

[34] Rubyogo J.C. (2004). https://cgspace.cgiar.org/handle/10568/70485.

[35] Kalyebara R., Buruchara R. (2008). http://ciat-library.ciat.cgiar.org/articulos_ciat/highlight41.pdf.

[36] Rwanda HarvestPlus. (2015). https://assets.publishing.service.gov.uk/media/57a0899eed915d622c0002f1/HarvestPlus_CountryBrief_Rwanda.pdf.

[37] Dimara E., Skuras D. (2003). Adoption of agricultural innovations as a two-stage partial observability process. Agric. Econ..

[38] Ruzzante S., Labarta R., Bilton A. (2021). Adoption of agricultural technology in the developing world: a meta-analysis of the empirical literature. World Dev.

[39] Mitra-Ganguli T., Boyd K., Uchitelle-Pierce B., Walton J. (2019). Proceedings of the workshop “Biofortified food – working together to get more nutritious food to the table in India. J. Nutr. Intermed. Metab..

[40] MINAGRI (2023). http://esoko.gov.rw/.

[41] Alioma R., Zeller M., Ling Y.K. (2022). Analysis of long-term prices of micronutrient-dense and starchy staple foods in developing countries. Agric. Food Econ..

[42] Dulleck U., Kerschbamer R., Sutter M. (2011). The economics of credence goods: an experiment on the role of liability, verifiability, reputation, and competition. Am. Econ. Rev..

[43] Pérez Suárez S. (2019). http://opus.uni-hohenheim.de/volltexte/2019/1637/pdf/DissertationSPerez.pdf.

