# Supplementary material for: Household-Level Coverage of Iron-Biofortified Beans in the Northern Province of Rwanda
Source: Curr Dev Nutr. 2023 May 27;7(6):100106. doi: 10.1016/j.cdnut.2023.100106 (PMC10314227; doi:10.1016/j.cdnut.2023.100106)
Supplement: Multimedia component 1 [file mmc1.docx]

**Supplemental Table 1.** Household coverage of iron-biofortified beans in Northern Province, Rwanda

| **Indicator** | **n** | **% (95% CI)** |
| --- | --- | --- |
| Household consumes beans in any form |  |  |
| Yes | 524 | 97.9 (96.4, 99.0) |
| No | 11 | 2.1 (0.1, 0.4) |
| Household is aware of iron-biofortified beans^1^ |  |  |
| Yes | 424 | 79.3 (75.6, 82.6) |
| No | 111 | 20.7 (17.4, 24.4) |
| Where respondent first heard about iron-biofortified beans |  |  |
| Fellow farmers/farmer groups | 188 | 35.1 (31.1, 39.4) |
| Radio/TV | 106 | 19.8 (16.5, 23.4) |
| Relatives/friends/neighbors | 81 | 15.1 (12.2, 18.5) |
| Extension agents/community health workers | 33 | 6.2 (4.3, 8.6) |
| Agro-dealers | 17 | 3.2 (1.9, 5.0) |
| Iron-biofortified beans are available in the household^2^ |  |  |
| Yes | 128 | 39.9 (34.5, 45.5) |
| No | 193 | 60.1 (54.5, 65.5) |
| Household ever consumed iron-biofortified beans^3^ |  |  |
| Yes | 216 | 51.9 (47.0, 56.8) |
| No | 192 | 46.2 (41.3, 51.1) |
| Household currently consumes iron-biofortified beans^4^ |  |  |
| Yes | 41 | 9.9 (7.2, 13.1) |
| No | 368 | 88.7 (85.3, 91.6) |

CI, confidence interval.

^1^ Households that do not consume beans in any form and households that reported not being aware of iron-biofortified beans were classified as “No.”

^2^ Only households that consume beans and provided bean samples were included in the analysis.

^3^ Households that do not consume beans in any form and households that never consumed biofortified beans were classified as “No.” Households that never heard of biofortified beans were excluded from analysis. Don’t know category (n=8, 1.9%) not presented.

^4^ Households that do not consume beans and households that did not consume biofortified beans in the past 7 days were classified as “No.” Households that never heard of biofortified beans were excluded from analysis. Don’t know category (n=7, 1.6%) not presented.

**Supplemental Table 2.** Household coverage of iron-biofortified beans by nutritional risk in Northern Province, Rwanda

|  | **Total** | |  | **Nutritional risk indicator** | | | | | | | | |
| --- | --- | --- | --- | --- | --- | --- | --- | --- | --- | --- | --- | --- |
| **Coverage Indicator** |  |  |  |  | **Adequate WDD Score** |  | **Inadequate WDD Score** |  |  | **Low MPI**  **(non-deprived)** | **High MPI**  **(deprived)** | |
|  | **n** | **% (95% CI)** |  | **n** | **% (95% CI)** | **n** | **% (95% CI)** |  | **n** | **% (95% CI)** | **n** | **% (95% CI)** |
| Household consumes beans |  |  |  |  |  |  |  |  |  |  |  |  |
| Yes | 524 | 97.9 (96.4, 99.0) |  | 253 | 97.7 (95.0, 99.1) | 271 | 98.2 (95.8, 99.4) |  | 227 | 99.1 (96.9, 99.9) | 297 | 97.1 (94.5, 98.6) |
| No | 11 | 2.1 (0.1, 4.0) |  | 6 | 2.3 (0.9, 5.0) | 5 | 1.8 (0.6, 4.2) |  | 2 | 0.9 (0.1, 3.1) | 9 | 2.9 (1.4, 5.5) |
| Household is aware of iron biofortified beans |  |  |  |  |  |  |  |  |  |  |  |  |
| Yes | 424 | 79.3 (75.6, 82.6) |  | **216** | **83.4 (78.3, 87.7)** | **208** | **75.4 (69.8, 80.3)** |  | **196** | **85.6 (80.4, 89.9)** | **228** | **74.5 (69.2, 79.3)** |
| No | 111 | 20.7 (17.4, 24.4) |  | **43** | **16.6 (12.3, 21.7)** | **68** | **24.6 (19.7, 30.2)** |  | **33** | **14.4 (10.1, 19.6)** | **78** | **25.5 (20.7, 30.8)** |
| Iron biofortified beans are available to the household |  |  |  |  |  |  |  |  |  |  |  |  |
| Yes | 128 | 39.9 (34.5, 45.5) |  | 65 | 38.0 (30.7, 45.7) | 63 | 42.0 (34.0, 50.3) |  | 65 | 40.4 (32.7, 48.4) | 63 | 39.4 (31.8, 47.4) |
| No | 193 | 60.1 (54.5, 65.5) |  | 106 | 62.0 (54.3, 69.3) | 87 | 58.0 (49.7, 66.0) |  | 96 | 59.6 (51.6, 67.3) | 97 | 60.6 (52.6, 68.2) |
| Household ever consumed iron biofortified beans |  |  |  |  |  |  |  |  |  |  |  |  |
| Yes | 216 | 51.9 (47.2, 52.0) |  | 101 | 47.6 (40.8, 54.6) | 115 | 56.4 (49.3, 63.3) |  | 98 | 50.5 (43.3, 57.8) | 118 | 53.2 (46.4, 59.9) |
| No | 192 | 46.2 (41.2, 50.9) |  | 106 | 50.0 (43.1, 56.9) | 86 | 42.2 (35.3, 49.3) |  | 91 | 46.9 (39.7, 54.2) | 101 | 45.5 (38.8, 52.3) |
| Don't know | 8 | 1.9 (0.8, 3.7) |  | 5 | 2.4 (0.8, 5.4) | 3 | 1.5 (0.3, 4.2) |  | 5 | 2.6 (0.8, 5.9) | 3 | 1.4 (0.3, 3.9) |
| Household currently consumed iron biofortified beans |  |  |  |  |  |  |  |  |  |  |  |  |
| Yes | 42 | 9.9 (7.2, 13.2) |  | 21 | 9.7 (6.1, 14.5) | 21 | 10.1 (6.4, 15.0) |  | 20 | 10.2 (6.3, 15.3) | 22 | 9.6 (6.1, 14.2) |
| No | 375 | 88.4 (85.0, 91.3) |  | 191 | 88.4 (83.4, 92.4) | 184 | 88.5 (83.3, 92.5) |  | 171 | 87.2 (81.7, 91.6) | 204 | 89.5 (84.7, 93.1) |
| Don't know | 7 | 1.7 (0.7, 3.4) |  | 4 | 1.9 (0.5, 4.7) | 4 | 1.4 (0.3, 4.2) |  | 5 | 2.6 (0.8, 5.9) | 2 | 0.9 (0.1, 3.1) |

Values in bold indicate significant difference between groups.

WDD: Women Dietary Diversity; MPI: Multi-dimensional Poverty Index.

**Supplemental Table 3.** Household coverage of iron-biofortified beans in relation to household socioeconomic characteristics

| **Socio-economic characteristics** | **Aware of IBBs** | **Ever consumed IBBs** | **Consumed IBBs (past 7 days)** | **IBBs available in the household** |
| --- | --- | --- | --- | --- |
| HH has a radio (yes vs. no) | **S** | **S** | **S** | NS |
| HH connected to electricity (yes vs. no) | **S** | NS | NS | NS |
| HH has a solar panel (yes vs. no) | **S** | NS | NS | NS |
| HH has a TV (yes vs. no) | Ns | NS | NS | NS |
| HH has a mobile phone (yes vs. no) | **S** | NS | NS | NS |
| HH has a bicycle (yes vs. no) | **S** | NS | NS | NS |
| HH has farmland (yes vs. no) | **S** | NS | NS | NS |
| Respondent education level (none or incomplete primary vs. complete primary or some secondary or higher education) | Ns | NS | NS | NS |
| Civil status (married vs. single/divorced/widowed | **S** | **S** | **S** | NS |

HH, Household; IBBs, Iron-biofortified beans; S, denotes significant at P< 0.05; NS, denotes not significant at P< 0.05.

Consumption of beans was not included as the expected count was < 5.
